# Legal duties, professional obligations or notional guidelines? Screening, treatment and referral of domestic violence cases in primary health care settings in South Africa

**DOI:** 10.4102/phcfm.v10i1.1724

**Published:** 2018-06-18

**Authors:** Lillian Artz, Talia Meer, Gray Aschman

**Affiliations:** 1 Gender, Health and Justice Research Unit, Division of Forensic Pathology, University of Cape Town, South Africa

## Abstract

**Background:**

Since 2013, approximately 4400 women have been murdered by their partners in South Africa. This is five times higher than the per capita global average. Domestic violence is known to be cyclical, endemic and frequently involves multiple victims. It also becomes progressively more dangerous over time and may lead to fatalities. In 2012, the Health Professions Council of South Africa released a domestic violence protocol for emergency service providers. This protocol, or screening guidelines, includes assessing future risk to domestic violence, providing physical and psychosocial care, documentation of evidence of abuse and informing patients of their rights and the services available to them. The extent to which these guidelines have been circulated and implemented, particularly by general health care practitioners (HCPs), is unknown.

**Aim:**

We review international treaties to which South Africa is a signatory, as well as national legislation and policies that reinforce the right to care for victims of domestic violence, to delineate the implication of these laws and policies for HCPs.

**Method:**

We reviewed literature and analysed national and international legislation and policies.

**Results:**

The ‘norms’ contained in existing guidelines and currently practiced in an ad hoc manner are not only compatible with existing statutory duties of HCPs but are in fact a natural extension of them.

**Conclusion:**

Proactive interventions such as the use of guidelines for working with victims of domestic violence enable suspected cases of domestic violence to be systematically identified, appropriately managed, properly referred, and should be adopted by all South African HCPs.

## Introduction

Sandile Mantsoe,^i^ Rameez Patel,^ii^ DJ Donal Sebolai,^iii^ Christopher Panayiotou,^iv^ Thato Kutumela^v^ and Oscar Pistorius^vi^ are all on trial or recently convicted for murdering their intimate partners in South Africa (SA). These cases received significant public attention, yet in the four-year span in which these notorious murders occurred, approximately 4400 women (three women daily) were also murdered by their partners in SA^1^ – five times higher than the per capita global average – but these deaths remain unacknowledged in public discourse and media. In 2015-2016, 275 536 domestic violence (DV) protection order applications were made.^2^ Though the Department of Health does not adequately record statistics relating to the presentation of DV cases in emergency medical care settings, DV is well documented as a public health issue. It is also recognised as a social and environmental risk to health and well-being, costing the country millions of rands annually in direct costs of disabilities and deaths and indirect costs of victims’ decreased work functionality, absenteeism and staff turnover.^3,4,5,6^ Whilst it is a form of violence that is known to be cyclical, endemic and frequently involving multiple victims, it is also progressively more dangerous over time and may lead to fatalities. A review of health sector initiatives in low and middle-income countries found that one of the key challenges to implementing successful DV interventions was health care systems’ lack of clear policies on DV; conversely, in places where policies, protocols and procedures are clear and well integrated, services for victims of DV became an effective part of routine health care.^7^

The Health Professions Council of South Africa’s (HPCSA) Annual Report for 2011-2012^8^ announced that: ‘at the start of 16 Days of Activism against [Violence against] Women and Children in 2011, the Board released the Domestic Violence Screening Protocol’ (p. 58). In 2012, Bateman^9^ wrote of emergency medical service providers’ concerns around implementing screening guidelines for emergency care workers handling victims of DV. He raised concerns about the extent to which DV screening guidelines could possibly address ‘all the variable and dynamic conflict scenarios’ (p. 343) in emergency medical contexts and the impact on already extremely high caseloads. Bateman^9^ referred to the over 2 million admissions to emergency centres annually, of which 40% (800 000) are trauma and injury, all excluding vehicle related injuries, in his appraisal of the viability of this protocol in practice (p. 343).

Bateman^9^ described the protocol as ‘generic high-level guidelines’ (p. 344) a set of procedures that include:

‘… assessing the risk and identifying imminent danger, providing supportive bio-psycho-social care, documenting diligently any evidence of abuse, informing patients of their rights, services and legal remedies, ‘talking through’ the implications of DV (including the risk of HIV) and referring responsibility appropriately whilst identifying their support systems.’ (p. 344)

The protocol also includes a diagrammatic representation of the screening process. In 2013, in the *HPCSA Bulletin*, Vinassa^10^ described the HPSCA protocol as: ‘a comprehensive set of universal screening guidelines to ensure that victims of abuse receive timeous and appropriate help’ and made the following observations:

‘Until the new guidelines were devised, there was no direct, unambiguous obligation for practitioners to screen routinely for abuse. Where, in the past, these practitioners were previously unprepared and poorly trained to deal with domestic violence, these guidelines will empower practitioners to intervene with the implementation of a prehospital protocol for domestic violence management.’ (p. 30)

Vinassa^10^ further noted:

‘The HPCSA’s professional Board for Emergency Care recognises that the right to health care intersects with the right to safety, and this intersection is apparent in the Health Professions Act, which indicates that practitioners have a duty to protect the safety of their patients.’ (p. 30)

An exhaustive Internet search, including of the HPCSA website, finds no evidence of the extent to which the protocol has been disseminated amongst emergency health care practitioners (HCP), and other HCPs, since their publication. In the absence of this, we reviewed international and regional (African) treaties to which SA is a signatory or whose norms have been incorporated into SA’s legal jurisprudence, as well as national legislation and policies that reinforce the rights of victims of DV, in order to delineate the implications for HCPs.

Through a qualitative systematic content analysis of these international and domestic laws and policies, it was found that these ‘norms’ are not only compatible with existing statutory duties of HCPs, but are in fact a natural extension of them. We further argue that proactive interventions, such as guidelines for working with victims of DV, not only enable suspected cases of DV to be identified, appropriately managed and properly referred, but that they may prevent the escalation of violence within domestic contexts and may go some way towards reducing the staggering levels of domestic homicide that South Africa is renowned for.

## International and national norms and obligations

The South African government has made significant commitments with regards to protecting victims of violence through the ratification of international instruments. In terms of Chapter 14, Section 232, of the *Constitution of South Africa* (1996), ‘…customary international law is law in the republic unless it is inconsistent with the Constitution or an Act of Parliament’. The breadth of both international laws signed and/or ratified by SA and the development of important domestic legislation and policy has not, however, resulted in consistent implementation in either the health or criminal justice systems. Yet, these international norms are regularly referred to and reflected on in key South African human rights literature and legislative developments. The more recognised of these are presented in Table 1.^11,12,13,14,15^

**TABLE 1:** International declarations.

International conventions	Key principles
*International Covenant on Civil and Political Rights* (1976) Ratified by SA in 1998^11^	An international declaration of human rights, in which the rights of women were specifically referenced for the first time.
*United Nations Declaration of Basic Principles of Justice for Victims of Crime and Abuse of Power* (1985)^12^ SA has been a member of the UN since 1945	Expressly recognises the rights of victims of DV in an international document. It sets out important principles for the treatment of victims of crime, including the rights to fair treatment, restitution, compensation and victim assistance. Section 14 states that victims (of crime) should receive the necessary material, medical, psychological and social assistance through governmental, voluntary, community-based and indigenous means. Section 15 explicitly states that victims should be informed of the availability of health and social services and other relevant assistance and be readily afforded access to these services.
*Convention on the Elimination of All Forms of Discrimination against Women* (CEDAW) (1979) Ratified by SA in 1995^13^	General Recommendation 19 of CEDAW sets out the duty of states to prevent and address VAW through, (1) preventive measures, including public information and education programmes to change attitudes towards the roles and status of men and women; (2) protective measures, including shelters, counselling, rehabilitation and support services for women who are the victims of violence or who are at risk of violence.
UN *Declaration of Elimination of Violence against Women* (1993) Ratified by SA in 1995^14^	Defines in detail what constitutes VAW, requiring states to condemn VAW and pursue by ‘…all appropriate means and without delay…’ a policy of eliminating VAW (Article 4). Its definitions of rape and DV have generally been adopted in SA’s DV and sexual offences laws.
The *United Nations Convention on the Rights of Children* (UNCRC) (1990) Ratified by SA in 1995^15^	The UNCRC contains 54 Articles pertaining to the human rights of children, including children’s rights to survival (Article 6), to protection from harmful influences (Article 32) and to protection against abuse and exploitation (Article 19). It has four founding principles: non-discrimination, best interests of the child, the child’s right to life and respect for the views of the child. South African child protection and child justice legislation has been significantly influenced by the UNCRC.

CEDAW, Convention on the Elimination of Discrimination against Women; DV, domestic violence; VAW, violence against women; UN, United Nations; UNCRC, United Nations Convention on the Rights of Children; SA, South Africa.

Note: Please see the full reference list of the article, Artz L, Meer T, Aschman G. Legal duties, professional obligations or notional guidelines? Screening, treatment and referral of domestic violence cases in primary health care settings in South Africa. Afr J Prm Health Care Fam Med. 2018;10(1), a1724. https:// doi.org/10.4102/phcfm.v10i1.1724, for more information.

Numerous international codes, including the World Health Organization’s (WHO) briefing document on injury prevention,^16^ propose the reduction of violence through victim identification, care and support programmes. WHOs policy guidelines *Responding to Intimate Partner Violence and Sexual Violence against Women: WHO Clinical and Policy Guidelines,*^17^ recommend screening when there are indicators of abuse, but (only) when patients’ privacy and safety can be ensured.

There are also several important regional instruments – including the Southern African Development Community’s (SADC) *Protocol on Gender and Development*,^18^ ratified by SA in 1997, and its accompanying *Addendum on Prevention and Eradication of Violence against Women and Children*,^19^ ratified in 2008 – that have emerged as more relevant instruments on violence prevention in the African context. The SADC protocol commits states to repealing and/or reforming all relevant laws and changing social practices that subject women to discrimination and violence. The addendum further commit states to recognising, protecting and promoting the human, reproductive and sexual rights of women and girls, as well as to taking measures to prevent and address the rising levels of violence against women (VAW) (Article. 20). The adoption of the *Protocol to the African Charter on Human and Peoples’ Rights on the Rights of Women in Africa*^20^ also marked a milestone in the protection and promotion of women’s rights in Africa by explicitly setting out the reproductive rights of women. The protocol mainly deals with VAW under two rights, the right to dignity (Article 3) and the right to life, integrity and security of the person (Article 4). State obligations to address VAW are specifically addressed under Article 4. Importantly, the protocol highlights the issue of sexual violence with respect to two groups of marginalised women – elderly women (Article 22[b]) and women with disabilities (Article 23[b]) – and states an undertake to ensure the freedom from violence of each group of women, including freedom from sexual abuse (Article 23[b]).

## South African legislation

Various South African laws and policies either provide opportunities for HCPs to screen patients for DV or in themselves imply DV screening. Such legislation includes the *Mental Health Care Act*, 2002 (Act No. 17 of 2002),^21^ the *National Health Act*, 2003 (Act No. 61 of 2003),^22^ the *International Health Regulations Act*, 1974 (Act No. 28 of 1974),^23^ the *Traditional Health Practitioners Act*, 2007 (Act No. 22 of 2007)^24^ and the *Choice on Termination of Pregnancy Act*, 1996 (Act No. 92 of 1996),^25^ with more focused violence prevention laws that enable health interventions including the *Domestic Violence Act* (DVA), 1998 (Act No. 116 of 1998),^26^ the *Children’s Act* (Act No. 38 of 2005)^27^ and the *Sexual Offences (and Related Matters) Amendment Act*, 2007 (Act No. 32 of 2007).^28,vii^ The HPCSA’s *Confidentiality: Protecting and Providing Information* policy (HPCSA, 2007),^29^ the *Guidelines for Good Practice in the Health Care Professions: General Ethical Guidelines for Reproductive Health*,^30^ the *Service Charter for Victims of Crime*^31^ and the *Minimum Service Standards for Victims of Crime*^32^ also invoke positive duties to intervene in domestic cases. We will provide the ambit and contents of these laws and policies below, with a particular emphasis on those that are most commonly applied in the intervention of DV.

Whilst it would seem sensible that the DVA^26^ – which aims to ensure that ‘…victims of DV receive the maximum protection from domestic abuse that the law could provide…’ (Preamble) – would be the genesis of health care responses to DV, it in fact includes no specifically prescribed duty for HCPs to intervene. It does, however, provide for victims to ‘obtain’ medical assistance. Like in many jurisdictions, the DVA is a law that fundamentally provides for protection orders that prohibit those who have been alleged to have committed act(s) of DV from committing further acts of violence. It does not criminalise DV per se, but instead criminalises the violation of the protection order. This legal remedy is meant to be preventative. As Aschman and colleagues^6^ note:

‘*The Domestic Violence Act* … does not impose any positive legal duties on HCPs to screen for DV, make referrals or holistically treat DV-related injuries and other health-related consequences of DV according to a medico-legal protocol. The Act merely implies that HCPs have a responsibility to attend to DV victims (section 2[a]) where it states that ‘any member of the SAPS must, at the scene of an incident of domestic violence or as soon thereafter as is reasonably possible, or when the incident of domestic violence is reported render such assistance to the complainant as may be required in the circumstances, including assisting or making arrangements for the complainant to find a suitable shelter and to obtain medical treatment.’ (p. 52–53)

However, in the HPCSA’s 2008 *Guidelines for Good Practice in the Health Care Professions: General Ethical Guidelines for Reproductive Health*,^30^ the HPCSA denounces VAW in the strongest terms and states that HCPs are uniquely placed to raise awareness of the problem (p. 5–6). Section 3 of these guidelines states that HCPs who treat female patients are ‘ethically obligated’ to inform themselves about the manifestations of violence and to ‘recognise cases’ (s. 31). Other obligations include treating the physical and psychological results of the violence (s. 3.1[i]), affirming to patients that violence is not acceptable (s.3.1[ii]) and advocating for social infrastructure to provide women (and presumably all victims of violence) the option of seeking secure refuge and ongoing counselling (s. 3.1[iii]). Section 3.2 further states that not redressing vulnerabilities to violence results in failure to prevent harm to future generations and contributes to the cycle of violence. On this basis, Section 3.2 provides that:

‘Practitioners treating women therefore have an obligation to, (i) affirm women’s rights to be free of physical and psychological violence, particularly sexual violence including sexual intercourse without consent within marriage, (ii) advocate for non-violent resolutions in relationships by enlisting the aid of social workers and other health care workers, where appropriate and (iii) make themselves and others, in particular men, aware of the harmful effects of the embedded discrimination against women in social systems.’

The *Mental Health Care Act*, 2002^21^ also contains several clauses that could reasonably be understood to pertain to DV screening and intervention. Section 11.1 defines responsibilities for ‘every person, body, organisation or health establishment providing care, treatment and rehabilitation services to a mental health care user’ including that they ‘must take steps to ensure’ that ‘users are protected from exploitation, abuse and any degrading treatment’. Such ‘exploitation’, ‘abuse’ and ‘degrading treatment’ surely includes DV. Section 34 of the Act also makes provision for a 72-h assessment and further involuntary care, treatment and rehabilitation. Such care, which involves observation and assessment, is also an opportunity to screen for and identify DV amongst mental health care users, especially given that DV and poor mental health, including mental health conditions, are often comorbid.^36^

Under the *Choice on Termination of Pregnancy Act*, 1996,^25^ anyone seeking an abortion should receive counselling, which would provide the opportunity for the counsellor to ask questions that may disclose DV or sexual assault. For example, where the Act stipulates that:

‘… in the case of a pregnant minor, a medical practitioner or a registered midwife, or registered nurse, as the case may be, shall advise such minor to consult with her parents, guardian, family members or friends before the pregnancy is terminated’ (s. 5.3) …

… an HCP could foreseeably discover abuse when inquiring why such a minor may choose not to disclose her pregnancy to others. Furthermore, given that there is ample evidence that pregnancy often leads to the onset or escalation of DV,^33,34^ screening young people and adults at this juncture may be crucial for maternal and foetal health.

The South African *Service Charter for Victims of Crime*^31^ was developed in line with SAs obligations under various international and regional human rights instruments and consolidates the current legal framework on the rights of victims of crime and the services that should be provided to them. Victims’ rights under the Charter include: the rights to fair treatment, access to information, protection, assistance, restitution and compensation. The principles governing the implementation of the Charter are laid out in the *Minimum Service Standards for Victims of Crime*.^32^ The Minimum Standards provide service practitioners with information on what is expected of them when rendering services to victims and provide clients with information on what to expect from practitioners, including HCPs.

## What does this mean for primary health care practitioners?

In the absence of specific guidelines to follow when a case of DV is identified, HCPs are often reluctant to screen for and treat DV.^6,35,37^ Health care practitioners providing services to victims of DV report feeling that intervening in DV is not their responsibility, that DV is too personal to discuss with patients, and that little can be done about preventing DV or helping victims.^38^ Health care practitioners find their lack of control over patients’ decisions frustrating and feel helpless when patients decide to remain in abusive relationships.^39^ Health care practitioners are also reluctant to engage with the legal process that may stem from a disclosure of DV, including testifying in court.^35,37^ Especially for HCPs involved in abusive relationships themselves, screening for DV amongst patients can be challenging.^38^ On a practical level, the under resourcing of health care facilities results in HCPs having insufficient time to screen and provide appropriate ‘extra medical’ services.^6^

Patients themselves are also sometimes reluctant to disclose DV because of denial, feelings of shame and lack of trust in HCPs. Victims often (justly) fear perpetrators’ reactions if they learn of the disclosure, and may fear that disclosure, even to non-legal service providers, will result in the involvement of the police, which may have several undesired consequences, such as the termination of the relationship, the break-up of the family and the loss of the primary breadwinner.^39^ Privacy, especially in primary health care settings, needs to be improved, as it would be unlikely for victims to disclose DV in the presence of other patients, and especially in the presence of perpetrators who may have accompanied them to the health care facility.^6^

Health care practitioners’ reluctance to screen and patients’ reluctance to disclose DV speak to the need for clear guidelines on where, when and how screening should take place, and specialised training on understanding DV, how to screen, how to encourage victims to disclose DV, and how to provide non-medical DV services and referrals. There are, of course, practical barriers to screening, including HCPs perceptions that screening for DV is invasive or ‘inappropriate’ or that it is not part of their health care provision obligations, the limited amount of time available to properly ‘build rapport’ before screening patients, apprehension of asking screening questions if domestic or intimate partner violence is not present in the patient’s life, and the lack of privacy in some health care settings.^6^ Indeed, professional and systematic barriers need to be considered before HCPs can be expected to feel competent in screening for DV. A small step towards this would be the provision of support from health care facilities in the process of adopting screening and referral measures, and in promoting DV screening as an important HCP responsibility.

## The possibilities of domestic violence screening, treatment and referral

Whilst there is no existing legislation and no well-defined policies that directly compel HCPs to screen for DV as a part of wider health care provision, such screening should become a routine part of providing adequate, preventative care under existing law. Read together, the ratified international and regional instruments, SAs national legislation and the HPCSA guidelines and protocols impose certain duties on the state, and all HCPs, to identify DV, inform patients about their rights and options, provide protective measures and, in the case of children, report abuse and maltreatment to a designated authority.

Several countries – including Spain, Finland, Serbia, Germany, the United States and Vietnam – have developed guidelines, policies and laws encouraging or obliging HCPs to screen and provide interventions for DV; therefore, the precedent for introducing similar policies and practices in SA has been set. Of particular interest are Serbia, categorised with SA as an upper middle-income country, and Vietnam, a lower-middle-income country. Under the *Special Protocol of the Ministry of Health of the Republic of Serbia on Protection and Treatment of Women Victims of Violence*,^40^ a manual for guiding HCPs in recognising and treating victims of gender-based violence was developed, and computer software was created to enable HCPs to document DV and its health repercussions in an electronic database, and to track the impact of these interventions. In Vietnam, the Hanoi Department of Health created the Improving Health Care Response to Gender-Based Violence project, introducing HCP training on routine screening of all female patients over the age of 15 and screening of girls younger than 15 if they showed symptoms of abuse.^41^ Services provided by the project include free counselling, voluntary counselling and testing for HIV and documentation of physical injuries without requiring that a charge be laid with the police. Subsequently, the Vietnamese government scaled up the project to all health care facilities throughout the country.

The International Planned Parenthood Federation (Western Hemisphere Region) ran a project to improve health care responses to gender-based violence, including DV, by working with local sexual and reproductive health care associations in the Dominican Republic, Peru and Venezuela.^42,43^ Standardised screening policies and protocols were implemented and new referral systems for counselling and legal assistance were developed. The rates of both screening and detection of gender-based violence increased in all participating facilities. Health care practitioners became more comfortable with conducting routine screening and came to see the value of screening, and clients interviewed as part of the programme evaluation reported feeling comfortable being asked screening questions. The acceptability of screening has also been found amongst women in a study conducted in Nigeria.^44^

The American Family Violence Prevention Fund also collaborated with two organisations in Mexico on gender-based violence screening programmes, both of which improved screening practices and increased DV-related interventions.^43^ In partnership with the non-profit organisation (NGO) *Alaide Foppa*, policies and protocols on screening and training were developed and implemented, referral networks were established and a public education campaign was run to encourage victims of gender-based violence to seek help at health care facilities. A second partnership with *Asesoría, Capacitación y Asistencia en Salud* worked with state HCPs and indigenous traditional birth attendants to intervene in abuse during pregnancy, to reduce morbidity and mortality of pregnant women and their children. The programme developed and implemented a DV screening protocol and trained state HCPs and traditional birth attendants on how to screen for and respond to DV amongst pregnant women.^43^ Similarly, the Prime II project in Armenia – which introduced a screening, treatment and referral programme in a reproductive health care clinic – resulted in improved HCP knowledge of and approaches to DV and increased the rate of detection of DV suffered by patients. Patients reported welcoming this assistance from the HCPs.^45^ Despite obvious challenges to state-based screening and intervention programmes, the prevention of DV is possible in developing contexts.

In SA, in 2003, Martin and Jacobs^46^ developed a strategic framework for use in state-run health care facilities to guide DV screening and holistic care of DV victims, which was designed to meet the objectives of both medical and psychosocial care, and evidence collection for criminal justice purposes. The framework includes a full suite of tools, a universal screening questionnaire, guidelines for HCPs on providing comprehensive physical and psychological care after DV disclosures, conducting safety assessments and developing safety plans with patients, providing patients with information about their legal rights and referring patients to external services for further assistance, and a comprehensive examination form to guide HCPs in documenting past and present incidents of DV, including physical injuries. The framework emphasises the importance of inter-sectoral cooperation and the need for HCPs and health care facilities to establish relationships with police, social workers and NGOs to ensure successful referral networks.^6^

In 2009, Joyner^37^ tested this screening framework in two urban and three rural health care facilities in the Western Cape and found that the framework met international standards and that the screening tools were sound. This was evidenced by the fact that 75% of the sample returned for follow-up consultations,^37^ 76% of returning participants reported that they found the intervention useful,^37^ the majority of participants reported appreciating that someone had enquired about their situation and found the referral services very useful, and some women reported that the intervention had encouraged them to make life-changing decisions. After adjusting the screening questionnaire’s emotional care provision to better assess and record patients’ emotional and mental state and adding a tool to screen for mental health problems, Joyner developed a comprehensive model for assisting HCPs in identifying and managing DV in primary health care settings. It should be noted that Joyner ultimately recommended a targeted rather than universal screening approach; instead of screening every patient, Joyner recommended only asking about DV when patients present to primary health care facilities with symptoms that are indicative of DV, and reserving universal screening for specialised health care facilities, such as HIV and family planning clinics.^37^ Nonetheless, the tool is appropriate for both targeted and universal screening. Joyner and Mash^47^ then published a guide for HCPs on how to identify symptoms of possible DV amongst patients, how to ask screening questions to confirm DV and how to provide services to DV victims. The tools necessary for implementing screening and related services nationally are thus already in place, and ready for adoption by the Department of Health.^6^ We believe that the adoption of these tools will necessarily pave the way for the reduction of barriers, as the uptake of screening must be accompanied by HCP training, and the mandating of training and screening procedures will in themselves increase the validity of screening as part of health care provision amongst HCPs.

In addition to the successful policies and laws mandating or encouraging DV screening by HCPs coming out of developing countries, SA would not be starting from scratch. The screening tools already developed and tested by Martin and Jacobs^46^ and Joyner and Mash^47^ are ready for adoption, and the International Planned Parenthood Federation manual would be a useful guide for HCPs and health care facility managers. South Africa arguably has one of the most well-developed and entrenched medico-legal systems in Africa, as evidenced by services available to survivors of sexual offences and is, therefore, well equipped to learn from other countries’ successes and best practices and to introduce routine screening for DV.

## Conclusion

It is widely accepted that if victims of any form of gender-based violence report to any service sector, it will be to a health facility.^6^ Medical attention is sought by victims of DV from emergency or trauma rooms, outpatient centres and general hospital admissions, primary health care settings and other health facilities such as obstetrics and gynaecology units and voluntary counselling and testing centres, yet injuries and other consequences of DV-related trauma and abuse are rarely documented and treated as DV by attending HCPs.^6^ In clinical health settings, women tend to report the secondary effects of DV (gynaecological issues, unresolved physical injuries and acute or chronic symptoms such as abdominal pain, gastric complaints, headaches and fatigue, anxiety and depression, ‘unexplained’ injuries and so on) which can alert HCPs to the possibility of DV.^37^ Thus, primary health care services are an ideal place to detect DV in more private settings, as well as an appropriate site from which victims can be provided with information about referral options, support services and available criminal justice options. There is ample international and national legislation promoting the role of HCPs in intervening in and preventing future incidents of DV. Vinassa^10^ makes the important point that screening that occurs as part of routine health history taking provides an opportunity for early detection and observes that existing HCPSA guidelines are consistent with both primary health care policy and South African health-related legislation, not to mention accepted international guidelines. Treating only the physical manifestations of a form of violence that is recurring, and precipitously becomes more violent, is not an option.

## Footnotes

i Accused of killing his ex-girlfriend Karabo Mokoena in April 2017.

ii Accused of murdering his wife Feroz Patel in April 2016.

iii Accused of murdering his girlfriend Dolly Tshabalala in June 2015.

iv Accused of having his wife Jane Panayiotou murdered in April 2015.

v Accused of murdering his girlfriend Zanele Khumalo in April 2014.

vi Convicted of killing his girlfriend Reeva Steenkamp in February 2013.

vii The *Sexual Offences (and Related Matters) Amendment Act* (28) has detailed regulations for the compulsory HIV testing of persons accused of rape (Section 27), the right of rape survivors to receive Post-Exposure Prophylaxis and other treatments for STIs at public health facilities (Section 28). The Department of Health’s 2009 *Health Directives* guide HCPs in implementing the Act with respect to pre- and post-HIV-test counselling and the e-administration of post-exposure prophylaxis to rape survivors.
